# Cellular effects of splenectomy on liver regeneration after 70% resection

**DOI:** 10.3389/fcell.2025.1561815

**Published:** 2025-05-01

**Authors:** Andrey Elchaninov, Polina Vishnyakova, Elena Gantsova, Miroslava Chirkova, Victoria Karyagina, Larkin Anatoliy, Evgeniya Kananykhina, Maria Kuznetsova, Ibrahim Atabekov, Evgeny Karpulevich, Silachev Denis, Dmitry Trofimov, Dmitry Goldshtein, Timur Fatkhudinov, Gennady Sukhikh

**Affiliations:** ^1^ Laboratory of Growth and Development, Avtsyn Research Institute of Human Morphology of FSBI “Petrovsky National Research Centre of Surgery”, Moscow, Russia; ^2^ Research Institute of Molecular and Cellular Medicine, Peoples’ Friendship University of Russia (RUDN University), Moscow, Russia; ^3^ Laboratory of Regenerative Medicine, Institute of Translational Medicine, National Medical Research Centre for Obstetrics, Gynecology and Perinatology Named after Academician V.I. Kulakov of Ministry of Healthcare of Russian Federation, Moscow, Russia; ^4^ Information Systems Department, Ivannikov Institute for System Programming of the Russian Academy of Sciences (ISP RAS), Moscow, Russia; ^5^ Faculty of Biology and Biotechnology, National Research University Higher School of Economics, Moscow, Russia; ^6^ Laboratory of Molecular Research Methods, Institute of Reproductive Genetics, National Medical Research Centre for Obstetrics, Gynecology and Perinatology Named after Academician V.I. Kulakov of Ministry of Healthcare of Russian Federation, Moscow, Russia; ^7^ Laboratory of Cell Technologies, National Medical Research Centre for Obstetrics, Gynecology and Perinatology Named after Academician V.I. Kulakov of Ministry of Healthcare of Russian Federation, Moscow, Russia; ^8^ Laboratory of Stem Cells Genetics, Research Centre of Medical Genetics, Moscow, Russia

**Keywords:** spleen, liver, regeneration, macrophages, monocytes

## Abstract

**Introduction:**

Mammalian liver regeneration is a complex process, the regulation of which involves many mechanisms. The immune system has a pronounced influence on the course of reparative processes in mammals. The hepatic portal vein system provides a direct anatomical connection between the liver and the spleen ― the largest lymphoid organ in mammals. Accordingly, the spleen may have a direct effect on liver regeneration as a source of biologically active substances and migrating leukocytes. Specific mechanisms of such influence remain understudied. This study aimed to assess the effect of splenectomy on liver regeneration after 70% resection in mouse model.

**Methods:**

Murine model of liver regeneration after 70% resection was reproduced in C57BL/6 male mice, some of them splenectomized 7 days before the liver resection. Proliferation marker Ki67 in the liver was assessed by immunohistochemistry and the protein content for cyclin D1, cyclin A2 and p53 in the liver was assessed by Western blotting. Using TUNEL assay, an increase in the number of apoptotic cells was detected. The highest number of TUNEL+ cells was detected 1 day after liver resection, while the number of apoptotic cells in animals with prior splenectomy was significantly lower compared to animals with preserved spleen. The dynamics of Ly6C+ monocytes and Ly6G+ leukocytes were studied by flow cytometry. Macrophages were isolated from the regenerating liver using magnetic sorting for F4/80 and their gene expression profiles were analyzed using Clariom™ S Assay, mouse. Peripheral blood and splenic monocytes were isolated by magnetic sorting for CD115 and analyzed by Illumina HiSeq 2500 platform RNA sequencing. Migration of peripheral blood and splenic leukocytes to the regenerating liver was studied using allogeneic transplantation of cells derived from B10-GFP mice.

**Results and discussion:**

Animals splenectomized prior to the liver resection showed higher rates of cell proliferation along with higher content of р53 protein in the remnant organ. Splenectomy also correlated with decreased rates of Ly6C+ monocyte and Ly6G+ leukocyte migration. Macrophages in the regenerating liver were transcriptomically enriched for signaling pathways associated with monocyte migration, cell adhesion and cell death. As shown by the GFP+ leukocyte transplantation experiment, the leukocytes migrating to the regenerating liver are mainly of splenic origin. According to high-throughput sequencing data, these cells express high levels of cell adhesion molecules. The spleen has a significant effect on liver regeneration through secretion of biologically active substances and migrating leukocytes. Pre-splenectomy leads to a more pronounced liver damage response after 70% resection, as indicated by higher rates of cell proliferation, higher p53 protein content and cell death-associated signaling pathway activation.

## Introduction

Liver regeneration is a complex multi-stage process regulated by many factors (Campana et al., 2021) mostly produced by the organ itself, with the exception of thyroid and adrenal hormones, as well as the epidermal growth factor EGF produced in Brunner glands of the duodenum (Michalopoulos and Bhushan, 2021). Among anatomical structures outside the liver thought to influence liver regeneration, spleen is a special focus, since the two organs are directly connected via splenic vein which belongs to the hepatic portal vein system (Elchaninov et al., 2022; Tarantino et al., 2021). The influence is presumably reciprocated via systemic circulation; in particular, the feedback may involve hepatocyte decay products that promote activation of various leukocyte populations in the spleen (Elchaninov et al., 2022; Tarantino et al., 2021).

Several mechanisms of influence of the spleen on the liver are considered. Most often, the spleen is considered as a possible source of synthesis of biologically active substances that can affect the liver. These substances include interleukins (Il1b, Il6, Il10) and growth factors (TGFβ, HGF) (Morinaga et al., 2010; Yin et al., 2011). Previously, we hypothesized a key role for inhibitors of serine and cysteine proteases, the expression of which is significantly increased in the spleen after experimental liver resection (Elchaninov et al., 2023).

A different mechanistic concept considers the spleen as a deposit of various leukocyte subsets that can migrate to the injured liver. Such route has been demonstrated in myocardial infarction and ischemic stroke models (Swirski et al., 2009; Kim et al., 2014). At the same time, growing tumors are colonized by blood monocytes that has not been deposited in the spleen (Shand et al., 2014). For liver repair, corresponding data are missing.

The third mechanistic concept considers the possible influence of the spleen on hemodynamic conditions in the liver. Liver resection causes an increase in the portal vein blood pressure (Michalopoulos and Bhushan, 2021) and signs of portal hypertension are also found in liver cirrhosis (Li et al., 2017). As the splenic vein is a major tributary of the hepatic portal vein, splenectomy can substantively reduce the blood pressure in this system (Li et al., 2017). A recently suggested additional link to liver homeostasis is indirect, involving splenic influence on the gut microbiota (Jin et al., 2024).

Although the hypothetical mechanisms of hepatosplenic influence have rather long been suggested, their actual contribution to liver regeneration remains unclear.

Liver macrophages are key accessory cells of the liver in terms of tissue homeostasis (Campana et al., 2021). Liver regeneration has been reported to involve both activation of resident liver macrophages and migration of monocytes to the liver. In this study we focus on splenic influence on macrophages in regenerating liver and more generally on splenic trace in the matter of leukocyte migration to the organ. As a model of liver regeneration, we used the compensatory growth of liver remnant after 70% resection in mice. This model represents an extensively studied variant of liver regeneration with a determined mass of lost parenchyma. To assess the splenic influence on liver repair, we used the splenectomy model, which is a clinically relevant choice. Splenectomy in patients with pathologically altered liver is often beneficial for the liver, but the mechanisms of the influence remain understudied.

Thus, for the first time, on a liver regeneration model after 70% resection in mice, the effect of splenectomy on the dynamics of liver mass restoration, proliferation dynamics, and the dynamics of the leukocyte population of the liver was studied. It was found that the spleen is a depot of leukocytes (granulocytes and monocytes), which migrate to the liver after its resection. Splenectomy reduces the level of granulocyte and monocyte migration to the resected liver.

## Materials and methods

### Animals

The study used C57BL/6 male mice, n = 62, body weight 20–22 g, purchased from Stolbovaya facilities (Russia).

The animals were kept in plastic cages at 22°C ± 1°C with light on from 6:00 a.m. to 6:00 p.m. and *ad libitum* access to standard food and water. The housing conditions complied with the International Recommendations for Conducting Biomedical Research on Animals of 1985 and the rules of laboratory activities in the Russian Federation: Order of the Ministry of Health of the Russian Federation No. 267 of 2003 and the “On the Protection of Animals from Cruelty” law, Chapter V, Article 10, 4679-GD, of 1999. The study was approved by the Ethics Committee of the Research Institute of Human Morphology named after A.P. Avtsyn (protocol No. 29 (5) of 8 November 2021). All procedures complied with ARRIVE guidelines.

### Model

**FIGURE 1:**
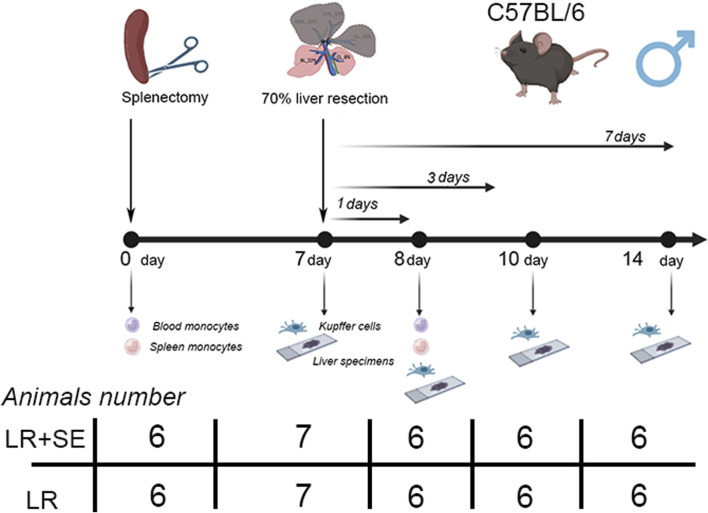
Scheme of experiment. LR – 70% liver resection, LR + SE - 70% liver resection in combination with previous splenectomy.

In the first series of experiments, liver resection was performed in intact animals (n = 25); in the second series of experiments the animals were splenectomized 7 days before the liver resection (n = 25). Sham-operated animals with intact liver (n = 6) and splenectomized animals with preserved liver (n = 6) were used as respective controls (Figure 1).

The interventions were carried out from 10.00 to 11.00 a.m. under general isoflurane anesthesia. In the postoperative period, the animals were housed two per cage under standard conditions.

For splenectomy, the abdominal cavity was opened, the spleen was brought into the wound and its vessels were ligated and then the spleen was completely removed; the wound of the anterior abdominal wall was sutured in layers. Liver resection with removal of 70% of the organ mass was performed according to the Higgins and Anderson method (Nevzorova et al., 2015). Splenectomy was performed 7 days before 70% liver resection. This protocol was chosen to allow experimental animals to cope with the stress caused by the operation. In clinical practice, splenectomy is performed simultaneously with liver resection. However, in our opinion, this operation to remove actually two organs is too traumatic for experimental animals. In addition, according to available data, the absence of the spleen continues to affect liver regeneration regardless of the period of asplenia (Babaeva, 1989). The ventral incision was closed with suture and treated with 0.05% chlorhexidine bigluconate followed by an alcohol swabbing. The anterior abdominal wall was sutured in layers. The muscles and skin were sutured with separate knotted sutures (COATED VICRYL® (polyglactin 910) Suture, Ethicon, United States). The level of pain was assessed by activity, food, and water intake. Meloxicam (1.0 mg/kg/day) was used to provide analgesia in the pre- and postoperative period and was administered 30 min before surgery and every 12 h for 2 days after surgery.

The animals were withdrawn from the experiment 24 h, 72 h and 7 days post-resection using a CO_2_ chamber.

### Histology and immunohistochemistry

Liver fragments were fixed in 10% neutral formalin and subjected to standard histological dehydration, paraffin embedding and sectioning. The 5–8 μm sections were stained with hematoxylin and eosin for pathomorphological assessment.

For immunohistochemical tests, the tissue was placed in liquid nitrogen and cryosectioned at 5–8 μm thickness. The sections were incubated with primary antibodies for 12 h and fluorophore-conjugated secondary antibodies (FITC or PE; 1: 200, Abcam, UK) for 1 h. Anti-Ki67 antibodies (ab15580, Abcam, UK) or Anti-caspase three antibodies (AF6311, Affinity Biosciences, UK) were used in 1:100 dilution; the nuclei were counterstained with 4′,6-diamidino-2-phenylindole (DAPI, Sigma-Aldrich Co LLC). The positivity indexes were determined as the percentage of labeled cells in total cell counts, with at least 3,000 cells counted for each marker.

### TUNEL-test

Test was performed using TUNEL assay kit-FITC (ab66108, Abcam, UK). Cell apoptosis was assessed by counting the number of TUNEL-positive cells (green) in the field with following normalizing to total cell number (red).

### Western blotting

The liver tissue was homogenized in a protein solubilization buffer (MicroRotofor Lysis Kit, BioRad, United States) supplemented with tributylphosphine and protease inhibitor cocktail. The homogenate was centrifuged at 14,000 g for 30 min. The supernatant was collected and mixed with precipitation buffer with added β-mercaptoethanol, heated for 5 min at +65°C and stored at −20°C. The proteins were separated by Laemmli electrophoresis in 10%–12.5% polyacrylamide gel and transferred to PVDF membranes (BioRad, United States) in Trans-Blot Turbo (BioRad, United States) at 0.35 A for 45 min. To block nonspecific antibody binding sites, the membrane was incubated in EveryBlot blocking buffer (BioRad, United States) for 40 min at room temperature. Incubation with antibodies to р53 (ab154036, Abcam, UK), Cyclin A_2_ (ab181591, Abcam, UK) or Cyclin D_1_ (ab134175, Abcam, UK) was carried out at +4°С overnight. After washing, the membranes were exposed to secondary HRP-conjugated antibodies for 1 h at room temperature. The chemiluminescence visualization was performed with Novex ECL kit (Invitrogen, United States) in a ChemiDoc imaging system (BioRad, United States). The signal intensity distribution analysis was performed using ImageLab software with GAPDH as normalizing protein. The uncropped Western blot membranes are presented in the Supplementary Material S1.

### Isolation of stromal cell fraction from the liver

Briefly, under anesthesia, the liver was perfused with a solution of phosphate-buffered saline via the portal vein. The organ was dissected, minced and incubated in 0.1% solution of type I and IV collagenases (PanEco, Russia). The suspension was passed through a 100 µm nylon strainer (SPL Life Sciences, Korea) and washed twice from the enzymes (300 g at 20°С for 10 min). The cells were resuspended in 30 mL of phosphate-buffered saline and centrifuged at 50 *g* for 3 min. As a result, parenchymal cells of the liver (hepatocytes) were sedimented and the non-parenchymal cell types (including liver macrophages, the Kupffer cells) remained in the supernatant.

### Isolation of liver macrophages

Separation of stromal cells from the bulk of hepatocytes was carried out as described in the previous paragraph. To enrich the stromal cell fraction with macrophages, the supernatant was mixed with Lympholyte®-M density separation medium (Cedarlane, Canada) and centrifuged at 400 *g* and 20°C for 30 min. The obtained fraction predominantly consisted of Kupffer cells. The suspension underwent immunomagnetic sorting on a manual MidiMACS™ Separator using LS Columns (Miltenyi Biotec, Germany) with Anti-F4/80 MicroBeadsUltraPure magnetic microparticles (Miltenyi Biotec, Germany) in accordance with the manufacturer’s recommendations. Each preparation contained pooled Kupffer cells from five mice.

### Isolation of monocyte-macrophage fraction from the spleen

Isolation the leukocyte fraction from the spleen has been described previously. Mechanical disaggregation, incubation with 0.05% solution of type I and IV collagenases (PanEco, Russia) and gradient centrifugation were used for this purpose (Elchaninov et al., 2023) The red blood cells were lyzed with Red Blood Cell Lysis Solution (Miltenyi Biotec, Germany); the total cell fraction was mixed with equal volume of Hanks’ solution supplemented with heparin (1000 IU/mL; Sintez, Russia). The mononuclear cell fraction was obtained by gradient centrifugation on Ficoll (PanEco, Russia; 400 g at 20°С for 30 min) The suspension underwent immunomagnetic sorting on a manual MidiMACS™ Separator using LS Columns (Miltenyi Biotec, Germany) with Anti-CD115 MicroBeadsUltraPure magnetic microparticles (Miltenyi Biotec, Germany) in accordance with the manufacturer’s recommendations. The obtained *monocyte-macrophage fraction* was immunophenotyped by flow cytometry.

### Isolation of monocytes from peripheral blood

Peripheral blood collected from male mice was mixed with equal volume of heparin-supplemented Hanks’ solution (1000 IU/mL heparin; Sintez, Russia). The mononuclear cell fraction was obtained by gradient centrifugation on Ficoll (PanEco, Russia; 400 g at 20°С for 30 min) and washed twice with Hanks’ solution (300 g at 20°С for 20 min. The suspension underwent immunomagnetic sorting on a manual MidiMACS™ Separator using LS Columns (Miltenyi Biotec, Germany) with Anti-CD115 MicroBeadsUltraPure magnetic microparticles (Miltenyi Biotec, Germany) in accordance with the manufacturer’s recommendations.

### Model of splenic leukocyte migration to regenerating liver

The leukocyte fraction was isolated from the spleen of male B10-GFP mice by the protocol described in previous paragraphs. The B10-GFP male mice, body weight 20–22 g, received from the “Andreevka” branch of the Federal State Budgetary Institution of Science “Scientific Center for Biomedical Technologies of the Federal Medical and Biological Agency”. The B10-GFP mouse line is derived from a hybridization of C57BL/10SnY mice and C57BL/6TgN (ACTbEGFP)1Osb mice (Jackson Laboratory, United States).

The isolated cell fraction was infused to intact male C57BL/6 mice via tail vein, 8 × 10^5^ cells per animal, prior to 70% liver resection. The animals were withdrawn on days 1, 3 and 7 post-resection. The stromal fraction was isolated from the liver as described in previous paragraphs and the presence of GFP + cells was assessed by flow cytometry.

### Analysis of liver leukocyte subsets by flow cytometry

The stromal cell fraction of the liver was immunophenotyped for surface markers CD45, F4/80, CD11b, Ly6C, Ly6G. For immunostaining, 1 × 10^5^ cells were incubated in 100 µL Rinsing Solution (Miltenyi Biotec, Germany) with primary antibodies at room temperature for 1 h, washed, resuspended in phosphate-buffered saline and analyzed in a FACScan™ flow cytometer (Becton Dickinson, United States) with CellQuest software, 10^4^ cells per each measurement. The population of interest was defined in a forward-vs. side-scatter (FSC-SSC) plot to exclude debris. To assess the number of positive leukocytes, CD45^+^ cells were isolated in the SSC and CD45 dot plots and the positive subset was isolated in the SSC and F4/80|CD11b|Ly6C|Ly6G dot plots. The gating strategy is presented in the Supplementary Material S1. Gates for stained samples were set based on the autofluorescence of the unstained control sample for each group.

### Transcriptomics

Total RNA was extracted from cell preps using RNeasy Plus Mini Kit (QIAGEN, Germany). The Clariom™ S Assay, mouse (Applied Biosystems™, United States) used total RNA from liver macrophages sorted immunomagnetically for F4/80. The data have been deposited into the Gene Expression Omnibus (GEO) database, https://www.ncbi.nlm.nih.gov/geo (GEO accession: GSE266703).

The Illumina HiSeq 2500 sequencing used total RNA from peripheral blood and splenic monocytes obtained by magnetic sorting. The detailed procedure is available in the Supplementary Material S1.

The data have been deposited into the Gene Expression Omnibus (GEO) database, https://www.ncbi.nlm.nih.gov/geo (GEO accession: GSE263764).

### Enzyme-linked immunosorbent assay

The protein content measurements for C1INH (MEA235Mu, Cloud-Clone, United States), Cystatin A (SEA476Mu, Cloud-Clone, United States) and Serpina3g (EM0799, Fine Test, United States) in the blood were carried out using an enzyme-linked immunosorbent assay in accordance with the manufacturers’ recommendations.

### Statistics

The data were analyzed in SigmaStat 3.5 software (Systat Software Inc, United States) using One way ANOVA with Holm-Sidak *post hoc* test. Ki67 index data were compared using a z-test. The differences were considered statistically significant at *p* < 0.05. For pairwise comparisons, the method was linked to distribution type, with Student’s t-test used for normally distributed variables and Mann-Whitney U-test for distributions other than normal. The conformity of the distribution of signs to normality was checked using the Kolmogorov-Smirnov criterion.

## Results

### Characterization of the liver recovery process after 70% liver resection with regard to splenectomy status

**FIGURE 2:**
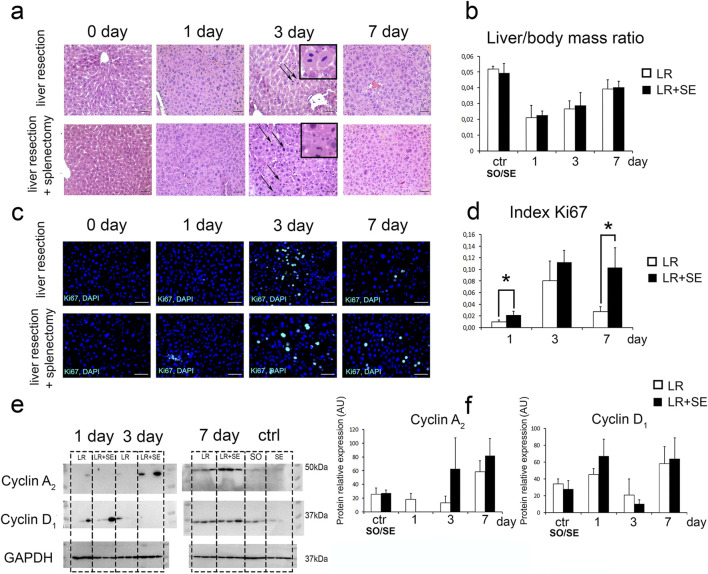
Characteristics of liver regeneration after 70% resection under conditions of previous splenectomy. **(a)** Histological structure of the regenerating liver, hematoxylin and eosin stain, scale bar - 100 μm, arrows indicate mitotic figures in hepatocytes. **(b)** Dynamics of liver mass recovery after 70% resection. The data were analyzed using One way ANOVA with Holm-Sidak *post hoc* test. The differences were considered statistically significant at *p* < 0.05. **(c)** Immunohistochemical study of the proliferation marker Ki67 in the regenerating liver. Second antibodies are conjugated to FITC (green), nuclei are counterstained with DAPI (blue), scale bars - 100 μm. **(d)** Dynamics of the Ki67 index, Ki67 index data were compared using a z-test. The differences were considered statistically significant at *p* < 0.05. **(e)** Western blot analysis of proteins associated with cell cycle (Cyclin A_2_, CyclinD_1_). **(f)** Densitometric study of protein content. Relative expression is presented as densitometry of protein bands in arbitrary units (AU). Data are presented as mean ± SD. The data were analyzed using t-test. The differences were considered statistically significant at *p* < 0.05. LR – 70% liver resection, LR + SE - 70% liver resection in combination with previous splenectomy, ctr - animals with an intact liver, black bar - splenectomized animals (SE) with preserved liver (n = 6) with the spleen removed 7 days before the experiment, white bar - sham-operated animals (SO) with intact liver (n = 6) with the preserved spleen, * - statistically significant differences, *p* < 0.05.

Mitotic figures in hepatocytes were recorded on days 3 and 7 post-resection in both groups (Figure 2a). Restoration of liver mass, the main indicator of the post-resection liver recovery, is complete in about 7 days post-resection (Figure 2b). The absence of the spleen did not affect the dynamics of liver mass restoration in experimental animals. Histological structure of the regenerating remnant liver in pre-splenectomized animals was similar to that in animals with preserved spleen (Figure 2b).

Between-the-group differences were found in the dynamics of proliferative activity in the regenerating liver. In animals splenectomized 7 days before the resection, Ki67+ cell index of the liver remnant was significantly higher (Figures 2c,e). Statistically significant differences were found 1 and 7 days after liver resection (Figures 2c,e). The resection caused an increase in cyclin A_2_ and cyclin D_1_ protein content of the liver (per unit volume) similar in both groups, i.e., independent of the splenectomy status (Figures 2e,f).

### Cell death dynamics

**FIGURE 3:**
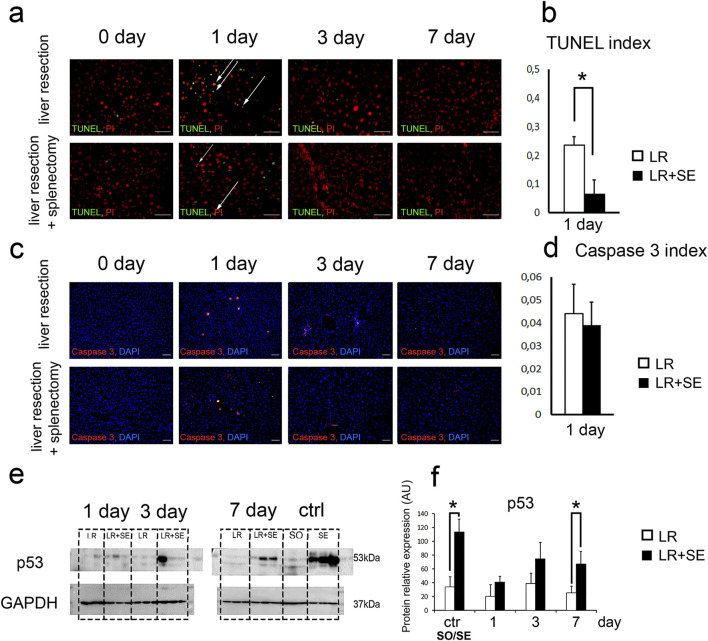
Cell death dynamics after 70% liver resection under conditions of previous splenectomy. **(a)** TUNEL-analysis. TUNEL+ nuclei are green. All nuclei are counterstained with PI (red). **(b)**. Dynamics of the TUNEL index. The data were analyzed using z-test. The differences were considered statistically significant at *p* < 0.05. **(c)**. Immunohistochemical study of the Caspase three in the regenerating liver. Second antibodies are conjugated to PE (red), nuclei are counterstained with DAPI (blue), scale bars - 100 μm. **(d)** Dynamics of the Caspase three index. The data were analyzed using z-test. The differences were considered statistically significant at *p* < 0.05. **(e)** Western blot analysis of proteins associated with cell death (p53). **(f)** Densitometric study of protein content. Relative expression is presented as densitometry of protein bands in arbitrary units (AU). The data were analyzed using t-test. The differences were considered statistically significant at *p* < 0.05. Data are presented as mean ± SD, LR – 70% liver resection, LR + SE - 70% liver resection in combination with previous splenectomy, ctr - animals with an intact liver, black bar - splenectomized animals (SE) with preserved liver (n = 6) with the spleen removed 7 days before the experiment, white bar - sham-operated animals (SO) with intact liver (n = 6) with the preserved spleen, * - statistically significant differences, *p* < 0.05.

According to the TUNEL analysis, the highest number of dying cells with DNA breaks was observed 1 day after liver resection (Figures 3a,b). Notably, a significantly higher number of apoptotic cells was detected in animals that underwent 70% liver resection compared to those that had a prior splenectomy (Figures 3a,b). However, when comparing the activity of caspase three at the same time point, no differences were found (Figures 3c,d). It is also noteworthy that a rather large number of small nuclei was observed among TUNEL+ cell nuclei, indicating the development of cell death in the cells of the liver stromal compartment (Figure 3a).

It is interesting to note that p53 protein expression in the liver tissue also increased in response to resection, and the increase was more pronounced in pre-splenectomized animals (Figures 3e,f). Statistically significant differences were found between animals with an intact liver, in which the spleen was preserved and splenectomy was previously performed, as well as in animals 7 days after liver resection (Figures 3e,f).

### Liver macrophages dynamics after 70% liver resection with regard to splenectomy status

**FIGURE 4:**
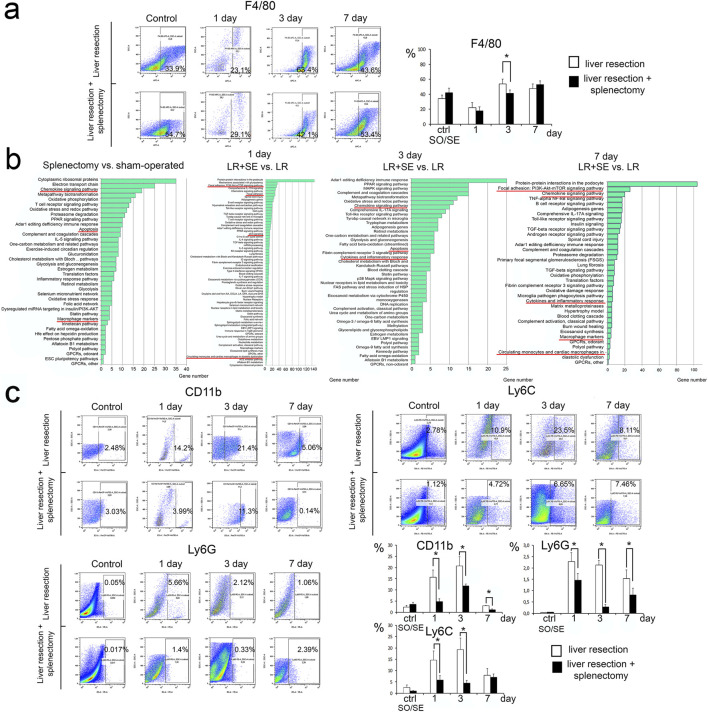
Characteristics of the leukocyte-macrophage system of the regenerating liver. **(a)** Dynamics of the population of F4/80+ macrophages in the regenerating liver. **(b)** Analysis of the transcriptome of macrophages in the regenerating liver. **(c)** Dynamics of monocytes and granulocytes. Data are presented as mean ± SD, LR – 70% liver resection, LR + SE - 70% liver resection in combination with previous splenectomy, ctr - animals with an intact liver, black bar - splenectomized animals (SE) with preserved liver (n = 6) with the spleen removed 7 days before the experiment, white bar - sham-operated animals (SO) with intact liver (n = 6) with the preserved spleen. The data were analyzed using t-test. The differences were considered statistically significant at *p* < 0.05, * - statistically significant differences, *p* < 0.05.

Splenectomy affects the leukocyte subsets of the liver, notably the monocyte-macrophage system dynamics during liver regeneration (Figure 4). Of note, the resection caused a decrease in differential counts of F4/80+ cells among the total fraction of liver stromal cells in both groups of the study (with and without splenectomy). Moreover, the number of F4/80+ cells in the liver was significantly lower in the group with prior splenectomy 3 days after liver resection (Figure 4a).

### Analysis of the transcriptome of macrophages from regenerating liver

Spleen removal influenced the gene expression profile of macrophages from both intact and regenerating liver. Signaling pathways indicating macrophage activation (*Chemokine signaling pathway, Macrophage markers*) as well as those associated with apoptosis were activated in macrophages from intact liver with prior splenectomy (Figure 4b). At 1 day after resection, signaling pathways also associated with cell death (*p53 signaling*) and cell adhesion and monocyte migration (*Focal adhesion: PI3K-Akt-mTOR signaling pathway, Focal adhesion, Circulating monocytes and cardiac macrophages in diastolic dysfunction*) were activated in F4/80+ liver macrophages after resection combined with prior splenectomy (Figure 4b). After 3 and 7 days, when comparing macrophages from regenerating liver from animals with preserved spleen and preliminary splenectomy, the most enriched signaling pathways were those related to macrophage activation (*Chemokine signaling pathway, Cytokines and inflammatory response, Macrophage markers*), activation of pathways related to apoptosis was preserved (Figure 3b). However, on the seventh day after resection among the enriched signaling pathways in macrophages the pathways related to cell adhesion and migration (*Focal adhesion: PI3K-Akt-mTOR signaling pathway, Circulating monocytes and cardiac macrophages in diastolic dysfunction*) appeared again (Figure 4b).

### Liver monocytes and granulocytes dynamics after 70% liver resection with regard to splenectomy status

After the resection, monocytes positive for Ly6C and CD11b markers migrate to the liver in high numbers. In pre-splenectomized animals, the number of monocytes migrating to the remnant liver was significantly lower, specifically for CD11b+ cells on day 1 and 3 post-resection, but not on day 7 post-resection (Figure 4c). A similar difference for Ly6C + cells (significantly fewer in splenectomized animals) was found only 3 days post-resection (Figure 4c). Pre-splenectomy also negatively affected the Ly6G + leukocyte infiltration of the liver in response to resection, with significant between-the-group differences for this marker observed on day 1 and 3 post-resection (Figure 4c).

### Migration of splenic leukocytes to regenerating liver

**FIGURE 5:**
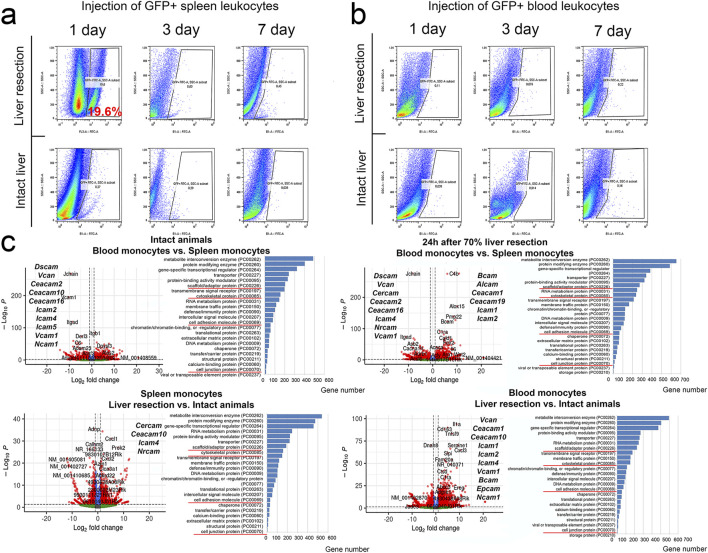
Detection of GFP + leukocytes from the spleen or peripheral blood in the regenerating liver. **(a)** Transplantation of GFP + spleen leukocytes. **(b)** Transplantation of GFP + blood leukocytes. **(c)** Transcriptome analysis of spleen and peripheral blood monocytes. The figure additionally shows genes with statistically significant altered expression associated with cell adhesion.

To demonstrate a fundamental possibility of the spleen leukocyte migration to regenerating liver, a suspension of splenic leukocytes from GFP + mice, all cells producing GFP, was infused to non-GFP mice immediately before the liver resection. As early as 24 h post-resection, the GFP + cells were found in the liver, constituting approximately 19% of all liver stromal cells (Figure 5a). When GFP + peripheral blood leukocytes were infused to animals after liver resection, no GFP + cells appeared in the liver (Figure 5b). When GFP + leukocytes isolated from the spleen or peripheral blood were infused to non-GFP mice with intact livers and no resection was carried out, no GFP + cells appeared in the liver of the recipient animals (Figures 5a,b).

### Comparative transcriptomic characterization of splenic and peripheral blood monocytes during liver regeneration

The resection had a broad effect on gene expression profiles in monocytes of peripheral blood and the spleen (Figure 5c). In peripheral blood monocytes, significant changes associated with the intervention were recorded for 4509 genes, including 2509 genes with increased expression and 2000 genes with decreased expression. The genes with increased expression belonged to 108 signaling pathways, most notably those involved in pro-inflammatory signaling mediated by chemokines (PANTHER list P00031), integrins (P00034), interleukins (P00036), cholecystokinin receptors (P06959) and cadherins (P00012) (Figure 5c).

Among genes activated in peripheral blood monocytes, the highest scores were observed for *Plexin*-B2 and *SH2D3C* associated with cell migration, chemokine *Cxcl3* and *Nos2* known as a marker of monocyte activation. Genes with decreased expression in peripheral blood monocytes (compared to the baseline levels) belonged to 96 signaling pathways and the majority was also associated with pro-inflammatory pathways P00031, P00034, P00036 and P06959 (Figure 5c).

In splenic monocytes, the intervention produced significant transcriptomic changes for 3,171 genes, including 1953 genes with increased expression and 1,218 genes with decreased expression (Figure 5c). The enrichment analysis identified 95 signaling pathways mostly associated with transcription and translation, as well as cytoskeletal dynamics, cell junction biosynthesis and cell adhesion molecule biosynthesis. Similarly with the data obtained for peripheral blood monocytes, the majority of genes annotated by the algorithm belonged to pro-inflammatory signaling pathways linked to chemokines, integrins, interleukins, and cholecystokinin receptors (respectively, P00031, P00034, P00036 and P06959). Genes with increased expression included a macrophage differentiation regulator PRKX, actin remodeling protein *CD2AP*, *Tns2* involved in cell migration and cell motility-associated *Memo1*. The genes with reduced expression belonged to a total of 70 signaling pathways with related biological implications (Figure 5c).

The discovered migration of infused splenic leukocytes to regenerating liver may be reflected by some transcriptomic features of these cells. Comparison of gene expression profiles for peripheral blood vs. splenic monocytes obtained from intact animals revealed decreased expression of CCR family genes, notably *Ccr8, Ccr3, Ccr6, Ccr10, Ccr1l1, Ccr1*, as well as *Cxcr1, Cxcr2, Vcam1* and *Stab2* involved in blood monocyte adhesion and motility, and increased expression of *Ccr9, Ccr4* and *Cx3cr1*.

The liver resection caused changes in expression of CCR family and other genes that regulate migration of both circulating and splenic monocytes. In blood monocytes, expression of *Ccr1, Ccrl2, Cxcr2* and *Cxcr1* increased, whereas expression of *Ccr4, Ccr9, Ccr7, Cxcr5* and *Cx3cr1* decreased after resection. In splenic monocytes, expression of *Ccrl2, Ccr7* and *Cxcr1* increased, whereas expression of *Ccr2, Ccr6, Cxcr6* and *Cx3cr1* decreased after resection. Comparison of post-resection profiles for blood vs. splenic monocytes revealed increased expression of *Ccr2, Ccr5, Ccrl2* and *Cx3cr1* and decreased expression *of Ccr3, Ccr10, Ccr1l1, Cxcr5* and *Cxcr4* in blood monocytes (Figure 5c).

### Dynamics of blood levels of protease inhibitors after liver resection

**FIGURE 6:**
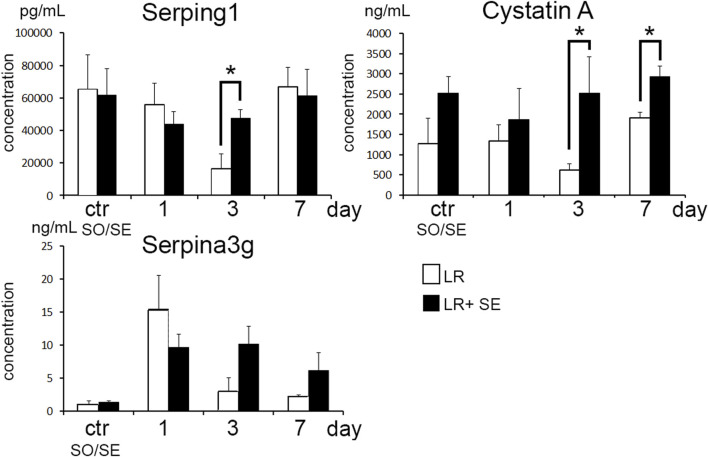
Dynamics of blood levels of protease inhibitors after liver resection. Data are presented as mean ± SD, LR – 70% liver resection, LR + SE - 70% liver resection in combination with previous splenectomy, ctr - animals with an intact liver, black bar - splenectomized animals (SE) with preserved liver (n = 6) with the spleen removed 7 days before the experiment, white bar - sham-operated animals (SO) with intact liver (n = 6) with the preserved spleen. The data were analyzed using t-test. The differences were considered statistically significant at *p* < 0.05, * - statistically significant differences, *p* < 0.05.

The resection promoted increase in blood levels for protease inhibitors Serping1 (C1INH), Serpina3g and Cystatin A in both groups (Figure 6). The dynamics was more pronounced in pre-splenectomized animals compared with the other group. Significant between-the-group differences were observed for Cystatin A on days 3 and 7 post-resection and for Serping1 on day 3 post-resection. A similar tendency for Serpina3g on days 3 and 7 post-resection was evident as well (respectively, *p* = 0.053 and *p* = 0.074 (Figure 6).

## Discussion

The results confirm the splenic influence in liver regeneration. In particular, pre-splenectomy affected the dynamics of proliferative response in the remnant liver after 70% resection in mice. Splenectomized animals had higher Ki67 proliferation index in the remnant liver compared to the control group. At that, relative mass of the regenerating liver in the two groups was similar.

Despite the unaffected liver mass recovery in splenectomized animals, the data arguably support a positive influence of healthy spleen on liver repair, at least in 70% liver resection model. Splenectomized animals presented with higher rates of cell death in regenerating liver, as indicated by increased content of p53 protein and activation of apoptosis/inflammation/chemokine-related signaling pathways in liver macrophages. The increased rates of cell death may promote a compensatory increase in proliferation activity; accordingly, the dynamics of liver mass recovery in splenectomized animals and animals with intact spleen are similar. However, we found that under splenectomy conditions, the level of cell death was lower in the resected liver compared to animals in which the spleen was preserved. This finding may be explained by the following: splenectomy affects systemic blood flow and through oxidative stress induces DNA damage which upregulates p53 production. p53 evokes DNA repair systems and consequently, we did not detect DNA damage according to TUNEL data, which suggests that this does not lead to the activation of cell death. Literature data indirectly support this concept (Lai et al., 2022). On the other hand, an increase in cell death can be observed at other time points after liver resection. Based on this, the mechanism underlying the activation of proliferation in the regenerating liver following prior splenectomy may involve not only the activation of cell death due to the lack of anti-inflammatory mediators supplied by the spleen (such as serine and cysteine protease inhibitors) but also the removal of hepatocyte proliferation blockers from the spleen (such as TGF-β) (Elchaninov et al., 2022; Elchaninov et al., 2020).

The assumed mechanisms of splenic influence on liver homeostasis involve cytokine secretion, leukocyte migration and portal hemodynamics (Elchaninov et al., 2022). An important functional link between the two organs involves the hemoglobin recycling cooperation (Mebius and Kraal, 2005). The capture of senescent red blood cells from circulation specifically occurs in the spleen. In splenic red pulp macrophages, heme uptake has been shown to directly stimulate expression of the marker transcription factor SPIC (Delaby et al., 2008; Ganz, 2016). Resident liver macrophages (Kupffer cells) are also implicated in heme utilization and also express the transcription factor SPIC (Bennett et al., 2021). Other experimental studies indicate that splenectomy can reduce the degree of liver cirrhosis due to its effect on the gut microbiota (Jin et al., 2024).

The modes of splenic influence depend on integrity status of the liver ― either violated by trauma or extensive surgery, or non-violated. Thus, in the case of intact liver, splenectomy does not affect monocyte and granulocyte populations of the liver, while affecting blood pressure in the portal vein system. Importantly, in splenectomized animals the heme neutralization burden is transferred entirely on liver macrophages, which is reflected by expression profiles of these cells.

The physical absence of the spleen modeled by splenectomy significantly affects the processes of monocyte and other leukocyte migration to the remnant liver after resection. The non-resident leukocyte contribution to the post-resection liver recovery has long been debatable (Michalopoulos, 2014), until accumulation of the data on granulocyte, lymphocyte and monocyte migration to the remnant liver (Nishiyama et al., 2015; Elchaninov et al., 2021). Here we also observe some infiltration of the remnant organ with Ly6C+CD11^+^ monocytes and Ly6G + granulocytes. The decreased rates of leukocyte infiltration of the remnant liver in splenectomized animals are consistent with the previously reported mild depletion of macrophages, monocytes and lymphocytes in the spleen after liver resection (Elchaninov et al., 2021). Signaling pathways associated with focal adhesion are activated in liver macrophages in response to resection, notably on day 1 post-resection; the subsequent recovery is marked by activation of inflammation-related signaling pathways in the remnant. The body of the evidence suggests that leukocytes migrating to the liver preliminarily accumulate in the spleen. The activation of signaling pathways associated with focal contacts and circulating monocytes indicates a compensatory reaction and may reflect the demand of the regenerating liver for colonization of the remnant by monocytes and other leukocytes.

The fundamental possibility of migration of splenic leukocytes into the regenerating liver was confirmed by us using a model of intravenous transplantation of GFP + splenic leukocytes from B10-GFP mice. At the same time, there was no migration of GFP + splenic leukocytes to the intact liver, or migration of GFP + peripheral blood leukocytes to the intact or regenerating liver. Thus, migration of leukocytes from the spleen is a specific process rather than mechanical transfer with the bloodstream through the splenic vein. That is, splenic monocytes are biologically different from peripheral blood monocytes in the context of post-resection recovery. The inference was confirmed by comparative transcriptomics of CD115+ splenic monocytes and peripheral blood monocytes. In intact animals, splenic monocytes express elevated baseline levels for a number of cell adhesion molecule genes including *Dscam*, *Vcan*, *Ceacam2*, *Ceacam10*, *Ceacam16*, *Icam2*, *Icam4*, *Icam5*, *Vcam1* and *Ncam1*. Resection of the liver promotes an increase in expression of cell adhesion molecule genes by peripheral blood monocytes. Accordingly, migration of these cells into the regenerating liver cannot be excluded, especially considering the increased expression of *Ccr2* gene involved in monocyte migration (She et al., 2022) by blood monocytes post-resection. Splenic monocytes, by contrast, show decreased *Ccr2* and *Cx3cr1* expression levels post-resection as compared to the baseline. These dynamics may indicate enhanced discharge of splenic monocytes into the bloodstream.

Biological significance of monocyte migration to the remnant liver should be addressed specifically. The resident liver macrophages constitute one of the most numerous macrophage populations in mammals (Liaskou et al., 2012). Under conditions of drastic loss in liver volume (modeled by 70% resection) the replenishment of liver macrophages may require extra cellular sources. Indeed, arrested monocyte entrance to the liver has been shown to slow down repair processes in the organ. On the other hand, resident macrophages and macrophages that differentiate from migratory monocytes may differ in properties, so that the latter may have some special role allocated to them in the context of recovery. This perspective is supported by studies showing that migratory monocytes and macrophages differentiating from them secure the restoration of the hepatic microvasculature (Zigmond et al., 2014; You et al., 2013).

In addition to monocyte migration, the general granulocyte marker Ly6G has indicated the migration of this type of leukocyte into the regenerating liver, as well as the effect of splenectomy on this process. The role of different types of granulocytes in liver regeneration is actively being studied. It has been shown that neutrophil migration is a crucial aspect of liver regeneration, as neutrophils stimulate the transition of macrophages—developing from monocytes that migrated to the liver—from a pro-inflammatory phenotype to an anti-inflammatory and pro-regenerative one (Yang et al., 2019). Furthermore, eosinophils secreting IL-4, which is necessary for initiating hepatocyte proliferation, are also essential for liver regeneration (Goh et al., 2013).

The further fate of monocytes that enter the regenerating liver is unclear. Most likely, the infiltrating monocytes and macrophages differentiating from them are gradually eliminated from the liver at the final stages of repair process, but the rates of elimination remain controversial. Some observations indicate that the number of migrated monocytes decreases already by 72 h after injury, while other show that this occurs only by day 14 (Nishiyama et al., 2015; Zigmond et al., 2014) This variation in estimates may be due in part to the markers used to identify monocytes. In particular, an increase in the number of CD11b+ cells in the liver after resection may reflect the induction of expression of this marker by resident liver macrophages; on the other hand, it may also reflect the simultaneous process of colonization of the organ by incoming monocytes. The rapid elimination of transplanted GFP + splenic cells (observed by us in the liver 24 h post-resection and absent at later stages) may be due to the allogeneic nature of the transplant.

Migration of monocytes to the liver can be associated with the death of resident macrophages in response to resection. The so-called macrophage disappearance reaction has been described in various models of damage to various organs including the liver. After damage to an organ, the number of resident macrophages decreases. Biological significance and molecular mechanisms of this phenomenon remain understudied. Death of resident macrophages by necroptosis or necrosis has been noted in bacterial and viral infections, as well as malaria (Blériot et al., 2015; DiPaolo et al., 2013; Lai et al., 2018). In the case of alveolar macrophages, the effect has been termed “defensive suicide” and regarded as initiatory damage required to trigger inflammatory responses, notably the recruitment of neutrophils and microbicidal monocytes, rather than a passive element of the infectious process. In this context, resident macrophages should be considered sensor cells rather than effectors (DiPaolo et al., 2013; Ginhoux et al., 2017).

The increased cell death rates among resident liver macrophages after massive resection of the organ may similarly support regeneration. The reduction in the number of resident macrophages per unit volume of tissue in the remnant organ observed by us in this study is consistent with our previous report on up to 16% cell death rates among F4/80+ liver macrophages after 70% liver resection in mouse model (Elchaninov et al., 2021). The present study detected activation of cell death-related signaling pathways in liver macrophages from animals that underwent splenectomy before liver resection. This is consistent with the flow cytometry data. Liver resection leads to a decrease in the number of F4/80+ macrophages in the liver among the stromal fraction cells. At the same time, 3 days after surgery, the number of F4/80+macrophages in case of preliminary splenectomy was significantly lower compared to animals with preserved spleen.

Several studies demonstrate increased expression of certain cytokines and growth factors (Il1, Il6, Il10, HGF, TGFβ, TNFα) in the spleen under conditions of liver damage (Kono et al., 1992; Ueda et al., 2003; Keramida et al., 2018). Our previous study demonstrates increased expression of protease inhibitors (Serpina3n, Stefin A2, Stfa2l1, Cathepsin G) by the spleen in response to 70% liver resection in experimental model (Elchaninov et al., 2023).

The role of protease inhibitors in regeneration is a rather new research focus. Induced overexpression of Serpina3n in paracetamol-induced hepatotoxic injury model alleviates the necrotic and inflammatory changes to the organ (Tran et al., 2023). A similar association was observed in experimental ischemic stroke (Zhang et al., 2022). The roles of Stefin A2 and Stfa2l1 in liver repair have not been studied, although Stefin A2 and its homologs are thought to alleviate the degree of inflammatory response by inhibiting cysteine peptidases, notably Cathepsins L and S involved in antigen presentation (Mihelič et al., 2006). Cathepsin S inhibition in Sjögren syndrome reduces the auto-antibody production and alleviates the lymphocytic infiltration of salivary and lacrimal glands (Roper et al., 2003; Saegusa et al., 2002). Removal of the spleen as a source of protease inhibitors can lead to a decrease in their blood levels. However, according to the immunosorbent assay, in pre-splenectomized animals plasma levels of Cystatin A (S*tefin A*) and C1-inhibitor (*Serping1*) after liver resection were higher compared to animals with intact spleen. Apparently, the absence of the spleen can aggravate the inflammatory reaction to liver resection which involves a compensatory increase in the production of these protease inhibitors outside the spleen.

We would like to point out some limitations of our study. First of all, it concerns transplantation of GFP + leukocytes derived from B10-GFP + mice. Formally, this is an allogeneic transplantation, which may affect the rate of their elimination from the donor’s body. However, it should be noted that the line of B10-GFP + mice is derived from C57BL/6, to which we transplanted isolated GFP + leukocytes. In our opinion, this should reduce the probability of rejection of the injected cells.

In addition, our study was performed on male C57BL/6 mice. We cannot exclude that the lineage of mice as well as the sex may be of importance in the conditions of our experiment. Liver regeneration has been a long studied problem in experiment. Since the beginning of the systematic study of liver regeneration in experiment, several factors influencing this process have been studied, including the influence of sex. Many studies have shown that castration leads to slower weight regeneration in laboratory animals. On the contrary, the introduction of sex hormones stimulates this process. There are contradictory data on the fact that liver regeneration is faster in those animals. Some studies show that in general females have faster liver regeneration compared to males (Birrer et al.; Liozner and Liozner, 1974). In other works, no difference in liver weight regeneration in females and males was found (Lee and Jung, 2023). In our opinion, the reason why not all studies show faster liver regeneration in females compared to males is that the recovery of liver mass after resection is extremely rapid. Within 7–10 days, the liver weight does not differ from the control.

## Conclusion

Splenectomy does not improve (may inhibit) liver regeneration after 70% resection, since the spleen is a source of leukocytes and biologically active substances, including protease inhibitors, entering the regenerating liver. The finding is contrary to some experimental studies demonstrating the positive effect of splenectomy on repair processes in the liver. The controversy may be due to the nature of the model liver injuries used in different studies. Under conditions accompanied by severe hepatocyte death, such as hepatitis or liver fibrosis, the spleen acts as additional source of damaging cytokines, so that its removal can stimulate reparative processes in the liver. Under conditions of liver resection, the rates of hepatocyte death in the remnant liver are minimal and no decay products reach the spleen via systemic circulation to promote excessive production of pro-inflammatory cytokines by the spleen. This explains the negative effect of splenectomy on the condition of the liver in normal conditions and during regeneration after 70% resection (Babaeva, 1989; Maruya et al., 2020).

## Data Availability

The datasets presented in this study can be found in online repositories. The names of the repository/repositories and accession number(s) can be found below: https://www.ncbi.nlm.nih.gov/geo/, GSE266703. https://www.ncbi.nlm.nih.gov/geo/, GSE263764.

## Abbreviations

DAPI, 4′,6-diamidino-2-phenylindole; GFP, green fluorescent protein; FITC, fluorescein isothyocyanate; PVDF, polyvinylidene fluoride; GAPDH, Glyceraldehyde 3-phosphate dehydrogenase.
