# Supplementary material for: Cellular effects of splenectomy on liver regeneration after 70% resection
Source: Front Cell Dev Biol. 2025 May 1;13:1561815. doi: 10.3389/fcell.2025.1561815 (PMC12078306; doi:10.3389/fcell.2025.1561815)

Cellular effects of splenectomy on liver regeneration after 70% resection

Andrey Elchaninov[1,3]*, Polina Vishnyakova[2,3], Elena Gantsova[1,3], Miroslava Chirkova[4], Victoria Karyagina[2], Larkin Anatoliy[5], Evgeniya Kananykhina[1], Maria Kuznetsova[6], Ibrahim Atabekov[4], Evgeny Karpulevich[4], Silachev Denis[7], Dmitry Trofimov[6], Dmitry Goldshtein[8], Timur Fatkhudinov[1,3], Gennady Sukhikh[2]

1 Laboratory of Growth and Development, Avtsyn Research Institute of Human Morphology of FSBI "Petrovsky National Research Centre of Surgery", Moscow, Russia

2 Laboratory of Regenerative Medicine, Institute of Translational Medicine, National Medical Research Centre for Obstetrics, Gynecology and Perinatology Named after Academician V.I. Kulakov of Ministry of Healthcare of Russian Federation, Moscow, Russia

3 Research Institute of Molecular and Cellular Medicine, Peoples' Friendship University of Russia (RUDN University), Moscow, Russia

4 Information Systems Department, Ivannikov Institute for System Programming of the Russian Academy of Sciences (ISP RAS), Moscow, Russia

5 Faculty of Biology and Biotechnology, National Research University Higher School of Economics, Moscow, Russia

6 Laboratory of molecular research methods, Institute of Reproductive Genetics, National Medical Research Centre for Obstetrics, Gynecology and Perinatology Named after Academician V.I. Kulakov of Ministry of Healthcare of Russian Federation, Moscow, Russia

7 Laboratory of Cell Technologies, National Medical Research Centre for Obstetrics, Gynecology and Perinatology Named after Academician V.I. Kulakov of Ministry of Healthcare of Russian Federation, Moscow, Russia

8 Laboratory of Stem Cells Genetics, Research Centre of Medical Genetics, Moscow, Russia

* Correspondence:
Andrey Elchaninov
elchandrey@yandex.ru

Uncropped membranes of the western blot

CyclinA2

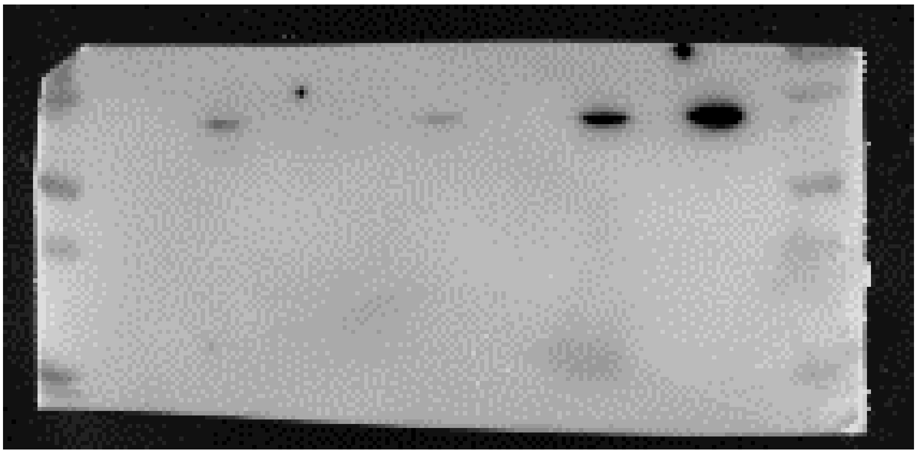

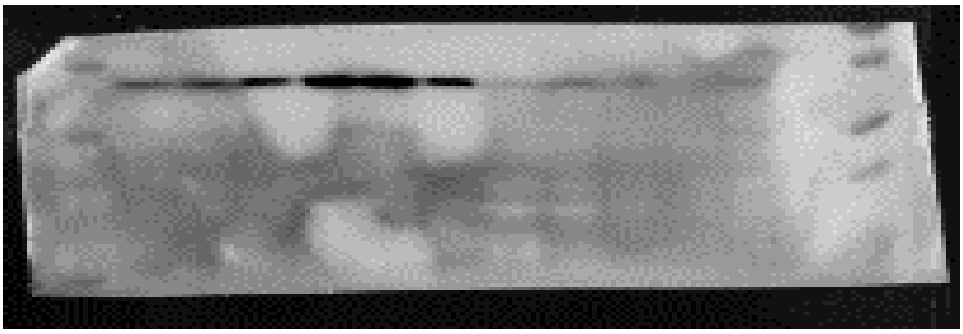

Cyclin D1

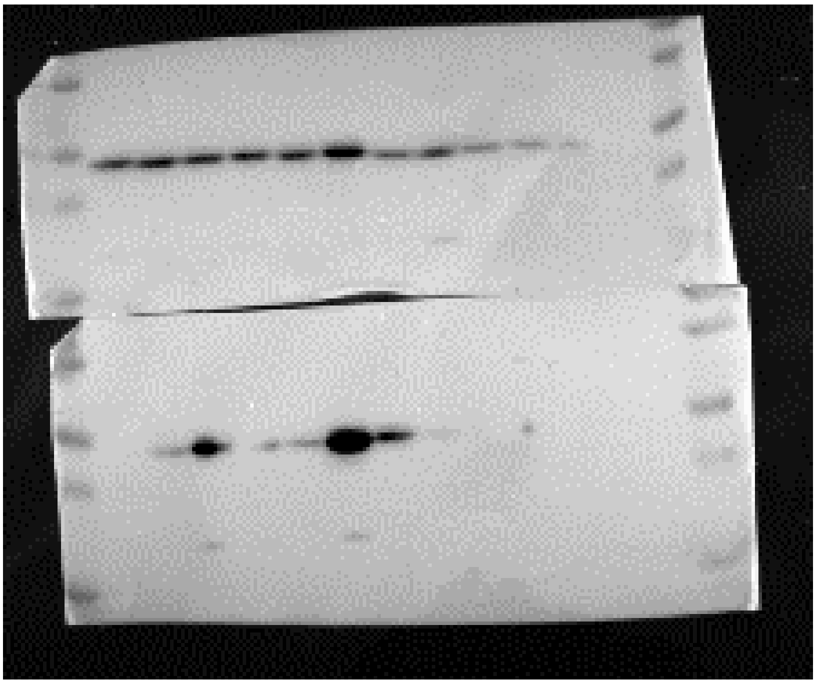

p53

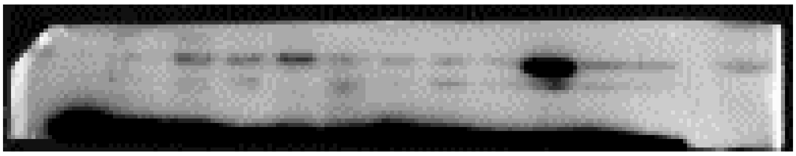

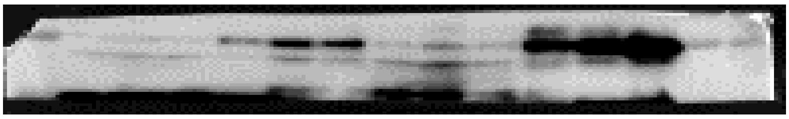

GAPDH

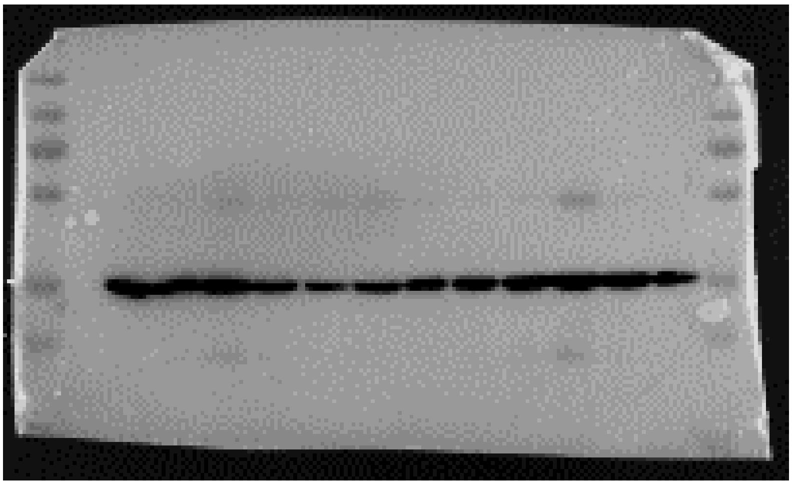

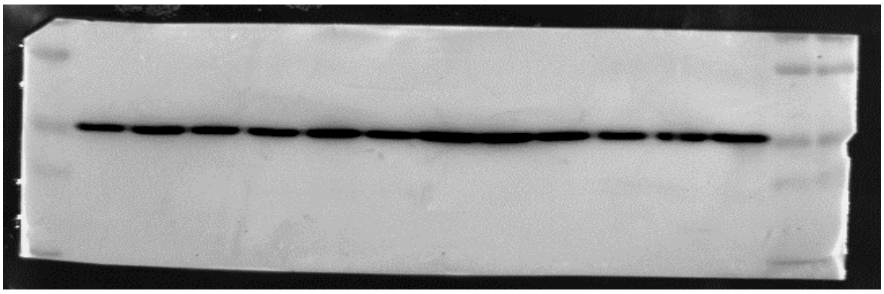

Supplement: Supplementary file 1 [file DataSheet2.pdf]
