# Supplementary material for: Cellular effects of splenectomy on liver regeneration after 70% resection
Source: Front Cell Dev Biol. 2025 May 1;13:1561815. doi: 10.3389/fcell.2025.1561815 (PMC12078306; doi:10.3389/fcell.2025.1561815)

Cellular effects of splenectomy on liver regeneration after 70% resection

Andrey Elchaninov[1,3]*, Polina Vishnyakova[2,3], Elena Gantsova[1,3], Miroslava Chirkova[4], Victoria Karyagina[2], Larkin Anatoliy[5], Evgeniya Kananykhina[1], Maria Kuznetsova[6], Ibrahim Atabekov[4], Evgeny Karpulevich[4], Silachev Denis[7], Dmitry Trofimov[6], Dmitry Goldshtein[8], Timur Fatkhudinov[1,3], Gennady Sukhikh[2]

1 Laboratory of Growth and Development, Avtsyn Research Institute of Human Morphology of FSBI "Petrovsky National Research Centre of Surgery", Moscow, Russia

2 Laboratory of Regenerative Medicine, Institute of Translational Medicine, National Medical Research Centre for Obstetrics, Gynecology and Perinatology Named after Academician V.I. Kulakov of Ministry of Healthcare of Russian Federation, Moscow, Russia

3 Research Institute of Molecular and Cellular Medicine, Peoples' Friendship University of Russia (RUDN University), Moscow, Russia

4 Information Systems Department, Ivannikov Institute for System Programming of the Russian Academy of Sciences (ISP RAS), Moscow, Russia

5 Faculty of Biology and Biotechnology, National Research University Higher School of Economics, Moscow, Russia

6 Laboratory of molecular research methods, Institute of Reproductive Genetics, National Medical Research Centre for Obstetrics, Gynecology and Perinatology Named after Academician V.I. Kulakov of Ministry of Healthcare of Russian Federation, Moscow, Russia

7 Laboratory of Cell Technologies, National Medical Research Centre for Obstetrics, Gynecology and Perinatology Named after Academician V.I. Kulakov of Ministry of Healthcare of Russian Federation, Moscow, Russia

8 Laboratory of Stem Cells Genetics, Research Centre of Medical Genetics, Moscow, Russia

* Correspondence:
Andrey Elchaninov
elchandrey@yandex.ru

Transcriptome analysis

The Illumina HiSeq 2500 sequencing used total RNA from peripheral blood and splenic monocytes obtained by magnetic sorting. The libraries were prepared from 1 µg total RNA using TruSeq RNA Sample Prep Kit v2 with 10 cycle enrichment step in accordance with the manufacturer's recommendations. The final libraries were pooled in equimolar ratios before sequencing in a 50 bp paired-end mode using Illumina HiSeq 2500 sequencing system. Raw reads were processed using RTA 1.17.21.3 and Casava 1.8.2 (Illumina). Processing of the initial data was carried out using Nextflow nf-core/rnaseq bioinformatics analysis to compare the control and one of the experimental groups. Commands for running the analysis of Blood/Blood, Spleen/Spleen groups:

./nextflow run nf-core/rnaseq --input ./mouse_rnaseq/blood_spleen.csv --genome Rnor_6.0 -profile docker

A csv file was passed as an input file (the "input" option) containing the paths to the fastq files of the samples of the two compared groups without chain specification ("strandedness" = unstranded). The reference genome M. musculus version Rnor_6.0 was retrieved from iGenomes [https://support.illumina.com/sequencing/sequencing_software/igenome.html] (the "genome" option) and docker [https://www.docker.com/] was used as a configuration profile (the "profile" option). Other parameters were set by default. The main steps of the pipeline are described in the documentation [https://nf-co.re/rnaseq/3.8.1]. Quantitative outputs for individual genes obtained by applying Salmon [Patro, 2017] to multiple alignments performed using the STAR tool [Dobin, 2013] were used in the Hobotnica pipeline [Stupnikov, 2021] to optimize the choice of tool for differential gene expression analysis. The tool selection algorithm [Stupnikov, 2021] promoted DeSeq2 as the fittest option; the results were filtered by a threshold of 0.1 for adjusted p-values.

1. Dobin, A., Davis, C. A., Schlesinger, F., Drenkow, J., Zaleski, C., Jha, S., ... & Gingeras, T. R. (2013). STAR: ultrafast universal RNA-seq aligner. Bioinformatics, 29(1), 15-2

2. Patro, R., Duggal, G., Love, M. I., Irizarry, R. A., & Kingsford, C. (2017). Salmon provides fast and bias-aware quantification of transcript expression. Nature methods, 14(4), 417-419.

3. Stupnikov, A., Sizykh, A., Budkina, A., Favorov, A., Afsari, B., Wheelan, S., ... & Medvedeva, Y. (2021). Hobotnica: exploring molecular signature quality. F1000Research, 10.

Gating strategy

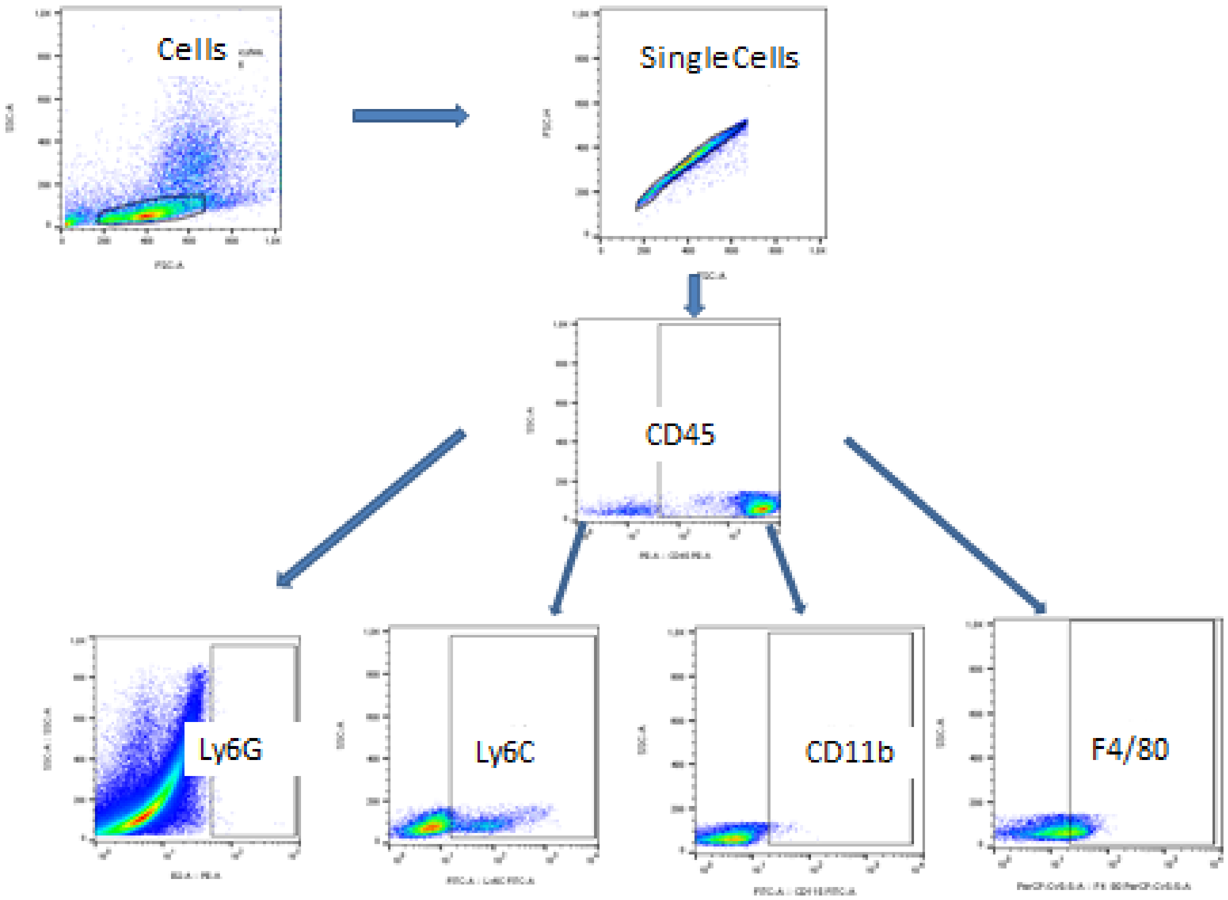

Supplement: Supplementary file 2 [file DataSheet1.pdf]
